# Methylene blue for intractable pain from oral mucositis related to cancer treatment: a randomized phase 2 clinical trial

**DOI:** 10.1186/s12916-022-02579-8

**Published:** 2022-11-03

**Authors:** Carlos J. Roldan, Billy Huh, Juhee Song, Yago Nieto, Joyce Osei, Thomas Chai, Kent Nouri, Lakshmi Koyyalagunta, Eduardo Bruera

**Affiliations:** 1 Department of Pain Medicine, Unit 409, The University of Texas MD Anderson Cancer Center, 1515 Holcombe Blvd, Houston, TX 77030 USA; 2 McGovern Medical School at the University of Texas Health Science Center at Houston (UT Health), Houston, TX USA; 3 Department of Biostatistics, The University of Texas MD Anderson Cancer Center, Houston, TX USA; 4 Department of Stem Cell Transplant, The University of Texas MD Anderson Cancer Center, Houston, TX USA; 5 The University of Texas MD Anderson Cancer Center, Houston, TX USA; 6 Department of Palliative, Rehabilitation, and Integrative Medicine, The University of Texas MD Anderson Cancer Center, Houston, TX USA

**Keywords:** Oral mucositis, Cancer therapy, Methylene blue Oral Rinse

## Abstract

**Background:**

Oral mucositis (OM) in patients receiving cancer therapy is thus far not well managed with standard approaches. We aimed to assess the safety and effectiveness of methylene blue (MB) oral rinse for OM pain in patients receiving cancer therapy.

**Methods:**

In this randomized, single-blind phase 2 clinical trial, patients were randomized to one of four arms: MB 0.025%+conventional therapy (CTx) (*n* = 15), MB 0.05%+CTx (*n* = 14), MB 0.1%+CTx (*n* = 15), or CTx alone (*n* = 16). Intervention groups received MB oral rinse every 6 h for 2 days with outcomes measured at days 1–2; safety was evaluated up to 30 days. The primary outcome measured change in the pain numeric rating scale (0–10) from baseline to day 2. Secondary outcome measured change in oral function burden scores from baseline to day 2, World Health Organization OM grades, morphine equivalent daily doses, and adverse events. The trial was registered with ClinicalTrials.gov ID: NCT03469284.

**Results:**

Sixty patients (mean age 43, range 22–62 years) completed the study. Compared with those who received CTx alone, those who received MB had a significant reduction of pain scores at day 2 of treatment (mean ± SD); 0.025%: 5.2 ± 2.9, 0.05%: 4.5 ± 2.9, 0.1%: 5.15 ± 2.6) and reduction of oral function burden scores (0.025%: 2.5 ± 1.55, 0.05%: 2.8 ± 1.7, 0.1%: 2.9 ± 1.60). No serious adverse events were noted, but eight patients reported burning sensation of the oral cavity with the first dose, and this caused one patient to discontinue therapy.

**Conclusions:**

MB oral rinse showed significant pain reduction and improved oral functioning with minimal adverse effects.

**Trial registration:**

ClinicalTrials.gov ID: NCT03469284.

**Supplementary Information:**

The online version contains supplementary material available at 10.1186/s12916-022-02579-8.

## Background

Oral mucositis (OM) is a debilitating condition that can occur in patients receiving oncologic therapy. OM initially manifests as oral mucosal erythema and mild pain. Still, it may progress to oral mucosal ulcerations and severe pain that affects oral intake, thus increasing morbidity, disrupting the quality of life, and adding to the cost of care. Risk factors for OM include the prior use of etoposide to mobilize peripheral blood progenitor cells, prior oropharyngeal radiotherapy, renal failure, poor performance status, and malnutrition [1].

Several agents have shown benefits for the prevention or treatment of OM. Intravenous palifermin showed effective prophylaxis compared with placebo in patients receiving cyclophosphamide/etoposide/total body irradiation [2] but not in patients receiving high-dose chemotherapy alone with melphalan [3] or in patients receiving busulfan/cyclophosphamide [4]. Similarly, amifostine was shown to be effective in preventing severe mucositis [5, 6]. Oral rinses with glutamine and supersaturated Ca^2+^ (PO_4_)_2_ both showed benefit in preventing and treating OM, but their effect in patients receiving stem cell transplantation appeared to be weak [7, 8]. Oral cryotherapy (e.g., ice chips) applied before, during, and shortly after the infusion of high dose melphalan is an inexpensive and effective preventive measure. However, its benefit is limited to this one drug, which is less extensively used chemotherapy [9]. Laser therapy appears to be effective in treating mucositis, but its use is limited by its logistic requirements [10].

In contrast, many interventions, some of which remain commonly used, are unproven or ineffective [11].

Although many efforts aim to resolve the histologic manifestations of OM, the biggest challenge in clinical practice is pain control [12]. Therefore, rather than studying the grades of severity and the healing time of OM, our efforts focused on pain management and its clinical implications. Methylene blue (MB) analgesic effect has been investigated more recently as a novel oral rinse for the management of oropharyngeal pain related to OM from cancer therapy. There is evidence in the literature to support MB for use as an analgesic for a variety of painful conditions [13–17]. Similarly, the safe and effective use of MB on OM have been published on retrospective studies [18, 19].

The purpose of this prospective, randomized trial was to determine the effectiveness and the safety of methylene blue oral rinse (MBOR) in the management of OM related to oncologic treatment.

## Methods

### Population

This was a prospective, randomized, single-blind, phase 2 clinical trial. Eligibility criteria included age of at least 18 years, a cancer diagnosis, receiving systemic chemotherapy, a current clinical diagnosis of OM, pain and oral dysfunction associated with OM despite conventional therapy (CTx), and voluntary written consent. Conventional therapy was defined as any intervention aimed at controlling oral pain, that included oral hygiene, analgesic rinses (Xylocaine; “magic mouth wash,” a compound mix of nystatin, hydrocortisone and diphenhydramine; compound diphenhydramine-antacids), and opiate analgesics. Exclusion criteria included known allergy to MB, pregnancy or breastfeeding, cognitive impairment and inability to consent, known history of G6PD deficiency, asymptomatic OM, and concomitant use of pro-serotonergic drugs. Patients undergoing radiation therapy were excluded due to the unknown ionization effect of MB on the radiating tissue.

The protocol met the criteria for an investigational new drug and was subject to federal regulations under the US Food and Drug Administration requirements. Therefore, specific parameters of safety report were imposed, and the use of a placebo was not allowed in this trial. It was also requested to test different concentrations of MB to find the lowest efficient dose. The protocol was approved by the institutional review board at The University of Texas MD Anderson Cancer Center, Houston, Texas. Trial registration: ClinicalTrials.gov ID: NCT03469284; protocol number: 2016-1051.

### Study setting and design

**Fig. 1:**
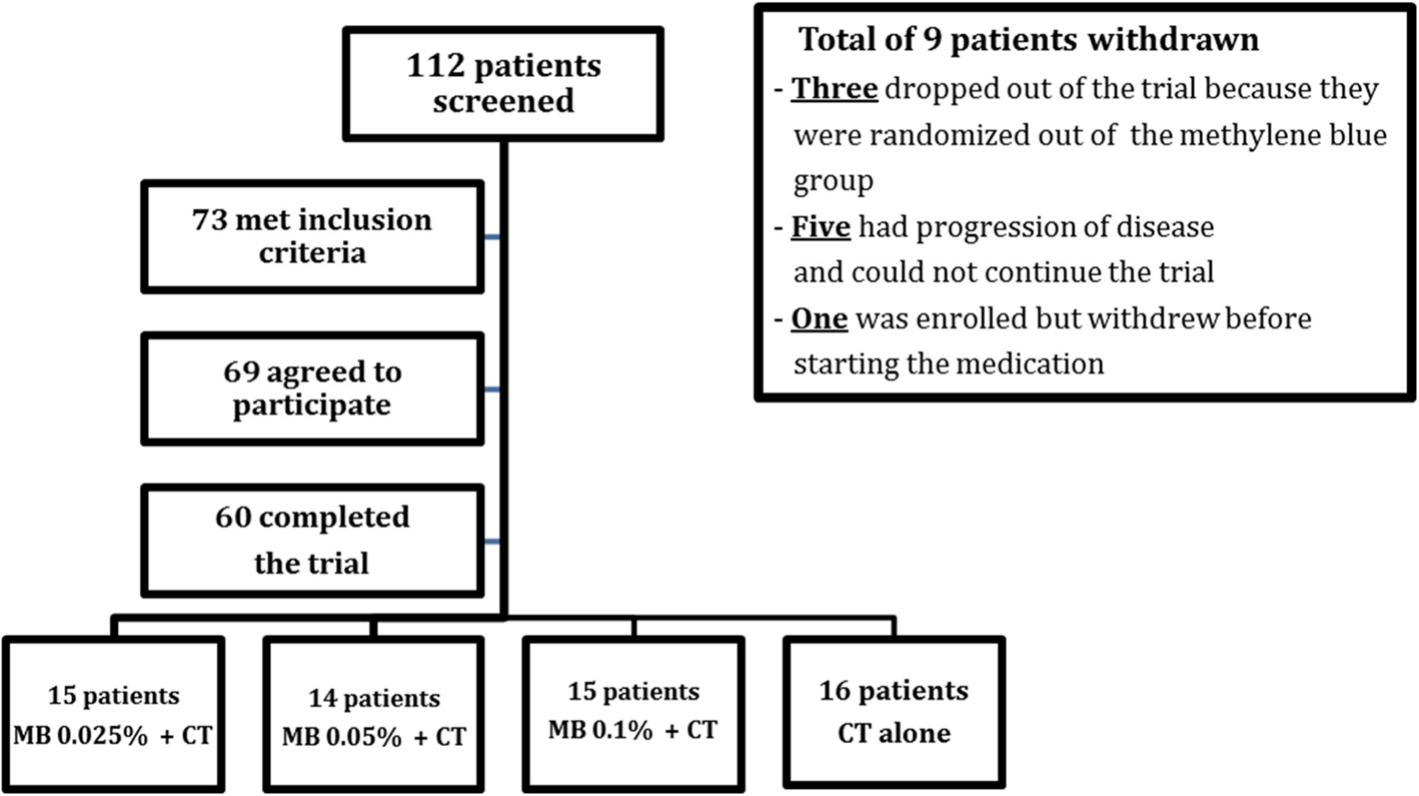
Flowchart of patients included in the trial. MB, methylene blue; CTx, conventional therapy

The study was conducted at The University of Texas MD Anderson Cancer Center involving adult patients with intractable pain associated with OM secondary to cancer therapy. Patients were enrolled between March 1, 2019, and December 01, 2020, and were randomized into one of four intervention arms: CTx alone (control arm) or 0.025%, 0.05%, or 0.1% MB solution in addition to CTx. Randomization allocation was downloaded from the institution’s clinical trial conduct website. All team members and patients were blinded to the concentration of MB used in each arm. MB was provided by the compound research pharmacy, responsible for the undisclosed dilution assigned. During the trial, three patients dropped out because they were disappointed at being assigned to receive CTx alone (Fig. 1).

### Endpoints and assessments

The primary goal of the trial was to evaluate the efficacy and safety of MBOR in reducing the severity of mucositis-related pain in cancer patients undergoing chemotherapy. The primary endpoint was the reduction in pain scores from baseline to 2 days, measured by the numeric rating scale (NRS), which ranged from 0 (no pain) to 10 (worst possible pain). Secondary endpoints included oral functioning burden (OFB; measured on a scale of 0, representing normal, to 6, meaning total inability, reflecting a total score of three categories: the ability to eat, swallow, and talk, each scored as unable = 2, difficult = 1, able = 0), morphine equivalent daily dose, and the incidence of adverse events after MB administration up to 30 days after the first dose of MB.

### Procedures

Once eligible, patients were randomly allocated to one of the MB treatment arms stratifying for baseline NRS and baseline OFB; the research pharmacy delivered a 100-mL solution bottle to the bedside. A research team member was present to provide final instructions and supervise the use of the first dose. Patients were instructed to take one mouthful (6–10 mL) of the MB solution and hold it at the painful sites for 5 min. Swishing and gargling were encouraged to reach all anatomical corners of the oral cavity. Patients were then instructed to spit and pause a few minutes before rinsing or ingesting meals. The same steps were repeated every 6 h (the total mix of 100 mL provided up to 6–10 uses or enough medication for about 2 days). Pain NRS and OFB scores were measured after each use and recorded at baseline and day 1, and day 2, after starting the treatment.

### Power calculation

For the primary outcome (OM pain reduction from baseline to 2 days after entering the study), we expected the mean pain reduction of 0, 2, 2.5, and 3 points for CTx alone, CTx + MB 0.025%, CTx + MB 0.05%, and CTx + MB 0.1% arms, respectively. The total sample of 60 subjects (15 in each arm) achieves 83% power to detect differences among the means versus equal means using an *F* test with a 0.05 significance level in a one-way ANOVA test. The size of the variation in the means is represented by their standard deviation which is 1.14 (means of 0, 2, 2.5, and 3 for CTx alone, CTx + MB 0.025%, CTx + MB 0.05%, and CTx + MB 0.1%, respectively). The common standard deviation within a group is assumed to be 2.5.

### Statistical analysis

Patient demographics and baseline characteristics, including baseline pain NRS scores and OFB scores, were summarized using descriptive statistics and compared by treatment group, utilizing Fisher’s exact test or chi-square test for categorical variables and Kruskal-Wallis test for continuous variables (CTx alone, CTx+MB 0.025%, CTx+MB 0.05%, and CTx+MB 0.1%). Changes in NRS and OFB scores from baseline to day 2 were compared among treatment arms using ANOVA. According to ANOVA test, pairwise comparisons (by Tukey method) were performed if the overall group showed significant differences. A *P*-value less than 0.05 indicated statistical significance. SAS 9.4 (SAS Institute INC, Cary, NC) was used for data analysis.

## Results

### Patient characteristics

Of 112 patients screened, 43 did not meet all inclusion criteria, 69 were enrolled, and 60 completed the trial (Fig. 1). Patients were recruited while hospitalized for various reasons and had various stages of OM. Patient clinical characteristics are summarized in Table 1. The mean age was 44 years (range, 19–76). There were 22 female and 38 male patients. The most common undergoing oncologic treatment was stem cell transplantation (*n =* 43, 72%), followed by systemic chemotherapy alone (*n =* 15, 25%). Most common therapeutic agents included carmustine/etoposide/cytarabine/melphalan combined, melphalan alone, vorinostat/gemcitabine/busulfan/melphalan mix, cyclophosphamide/etoposide combined, gemcitabine/docetaxel/melphalan/carboplatin combined, and etoposide alone.

**Table 1:** Patient characteristics by treatment arm

Covariate	Treatment arm, no. (%)				*P*^a^
	CT alone, *n* = 16	CT+MB 0.025%, *n* = 15	CT+MB 0.05%, *n* = 14	CT+MB 0.1%, *n* = 15	
Sex					0.9847
Male	10 (63)	10 (67)	9 (64)	9 (60)	
Female	6 (38)	5 (33)	5 (36)	6 (40)	
Age (in years), median (range)	48 (26–65)	45 (26–76)	43 (21–70)	32 (19–75)	0.5413
Race					0.8039
White	15 (94)	11 (73)	11 (79)	11 (73)	
Black	1 (6)	1 (7)	0 (0)	1 (7)	
Asian	0 (0)	1 (7)	1 (7)	1 (7)	
Other	0 (0)	2 (13)	2 (14)	2 (13)	
Therapy					0.2740
CAR-T	0 (0)	0 (0)	1 (7)	1 (7)	
Chemotherapy	3 (19)	2 (13)	6 (43)	4 (27)	
SCT	13 (81)	13 (87)	7 (50)	10 (67)	
Mucositis severity					0.1090
Grade 2	1 (6)	0 (0)	1 (7)	4 (27)	
Grade 3	15 (94)	15 (100)	13 (93)	11 (73)	
Mucositis location					0.8758
Oral mucosa	8 (50)	9 (60)	8 (57)	7 (47)	
Esophageal	8 (50)	6 (40)	6 (43)	8 (53)	
Baseline pain NRS score					0.9453
0–5	4 (25)	2 (13)	3 (21)	3 (20)	
6–10	12 (75)	13 (87)	11 (79)	12 (80)	
Baseline OFB score					0.6811
0–3	2 (13)	2 (13)	3 (21)	1 (7)	
4–6	14 (88)	13 (87)	11 (79)	14 (93)	
Pain duration (in days), median (range)	5.5 (3–8)	8 (2–90)	6 (1–43)	5 (2–30)	0.1046
OFB score^b^					
Baseline, median (range)	5 (2–6)	5 (2–6)	4.5 (2–6)	5 (2–6)	0.8428
Day 1, median (range)	4.5 (1–6)	3 (0–5)	2 (0–5)	2 (0–6)	0.0210
Day 2, median (range)	4 (0–6)	2 (0–5)	1 (0–5)	2 (0–5)	0.0008
Reduction at day 2, mean ± SD	0.81 ± 1.11	2.47 ± 1.55	2.79 ± 1.72	2.87 ± 1.60	0.0008
MEDD baseline, median (range)	117.5 (16.4–825)	115 (12.5–535)	107 (15–648)	120 (5–3153)	0.9182
Pain NRS score^c^					
Baseline, median (range)	6.5 (4–10)	8 (3–10)	7.5 (4–10)	8 (3–10)	0.1884
Day 1, median (range)	6 (2–9)	4 (0–8)	3 (0–7)	3 (0–8)	0.0052
Day 2, median (range)	5.5 (0–8)	3 (0–8)	2 (0–7)	2 (0–8)	0.0316
Reduction at day 2, mean ± SD	1.69 ± 3.09	5.2 ± 2.81	4.54 ± 2.93	5.15 ± 2.64	0.0034

*Abbreviations*: *CTx* conventional therapy, *MB* methylene blue, *CAR-T* chimeric antigen receptor T cell therapy, *SCT* stem cell transplantation, *NRS* numeric rating scale, *OFB* oral functioning burden, *MEDD* morphine equivalent daily dose

^a^Reductions in pain NRS and OFB scores from baseline to day 2 among four treatment arms were compared using ANOVA

^b^Scores ranged from 0 to 6

^c^Scores ranged from 0 to 10

The mucositis-related pain had lasted a median of 6 days (range, 1–90; IQR, 4–8) before the patient joined the trial. All patients had a clinical diagnosis of mucositis related to cancer therapy; per World Health Organization criteria, most patients had grade 3 mucositis (*n =* 54, 90%); the clinical severity of the OM was documented only at enrollment point to the study. All patients were actively receiving oral rinses and concomitant systemic opiate analgesics, with a median morphine equivalent daily dose (MEDD) of 113.75 (range, 5–3153). Most patients had severe OFB at baseline, with a median score of 5 (range, 2–6) on the 0–6 scale. No patients were reported to be receiving total parenteral nutrition.

All patients had painful lesions in multiple areas of the oral cavity. Many (*n =* 28, 47%) were suspected to have esophageal compromise; the oral mucosa was the most reported location (*n =* 39, 65%).

### Efficacy of MB

**Fig. 2:**
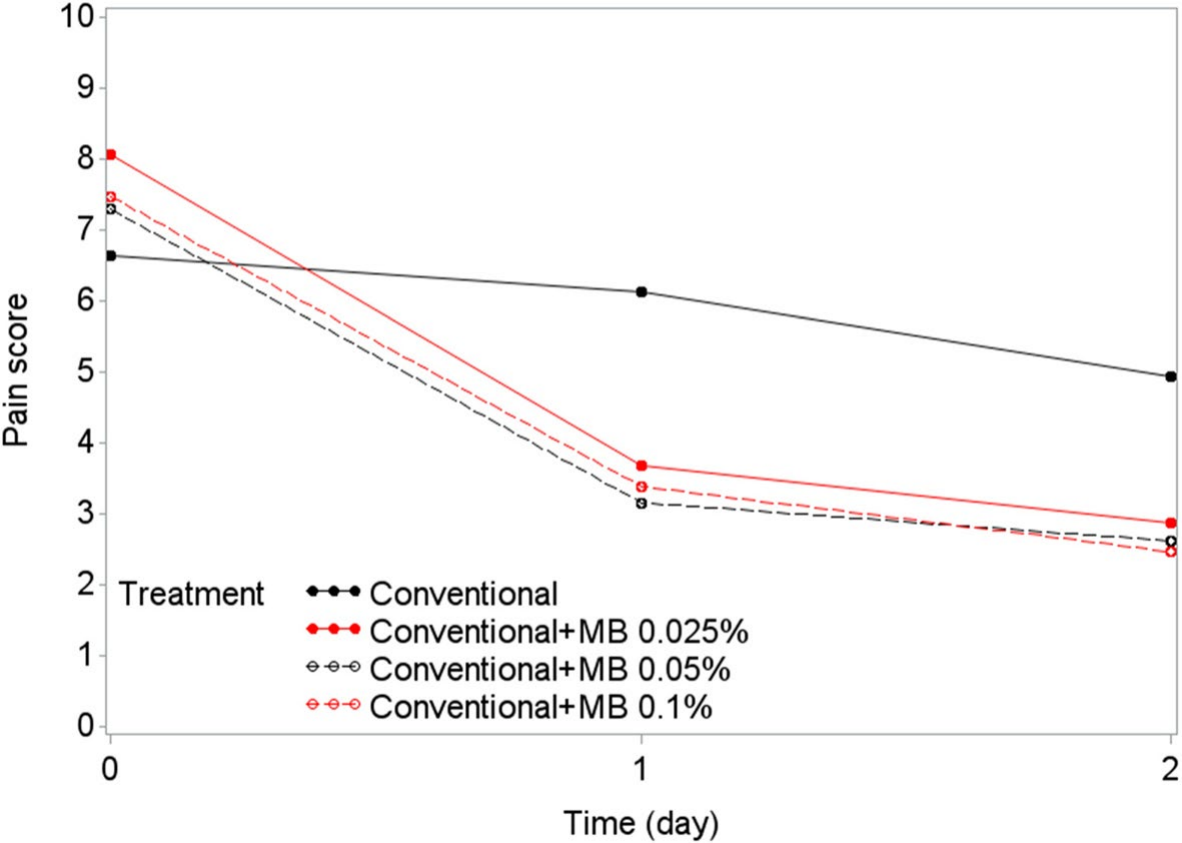
Changes in numeric rating scale (NRS) of pain scores over the first 2 days of treatment. Mean reduction in numeric pain scores at day 2 after the start of treatment were significantly or marginally better in patients who received conventional therapy plus methylene blue (MB) oral rinse at three different concentrations than in patients who received conventional therapy alone (Tukey pairwise comparison; *P* = 0.0071, *P* = 0.0506, *P* = 0.0114)

**Fig. 3:**
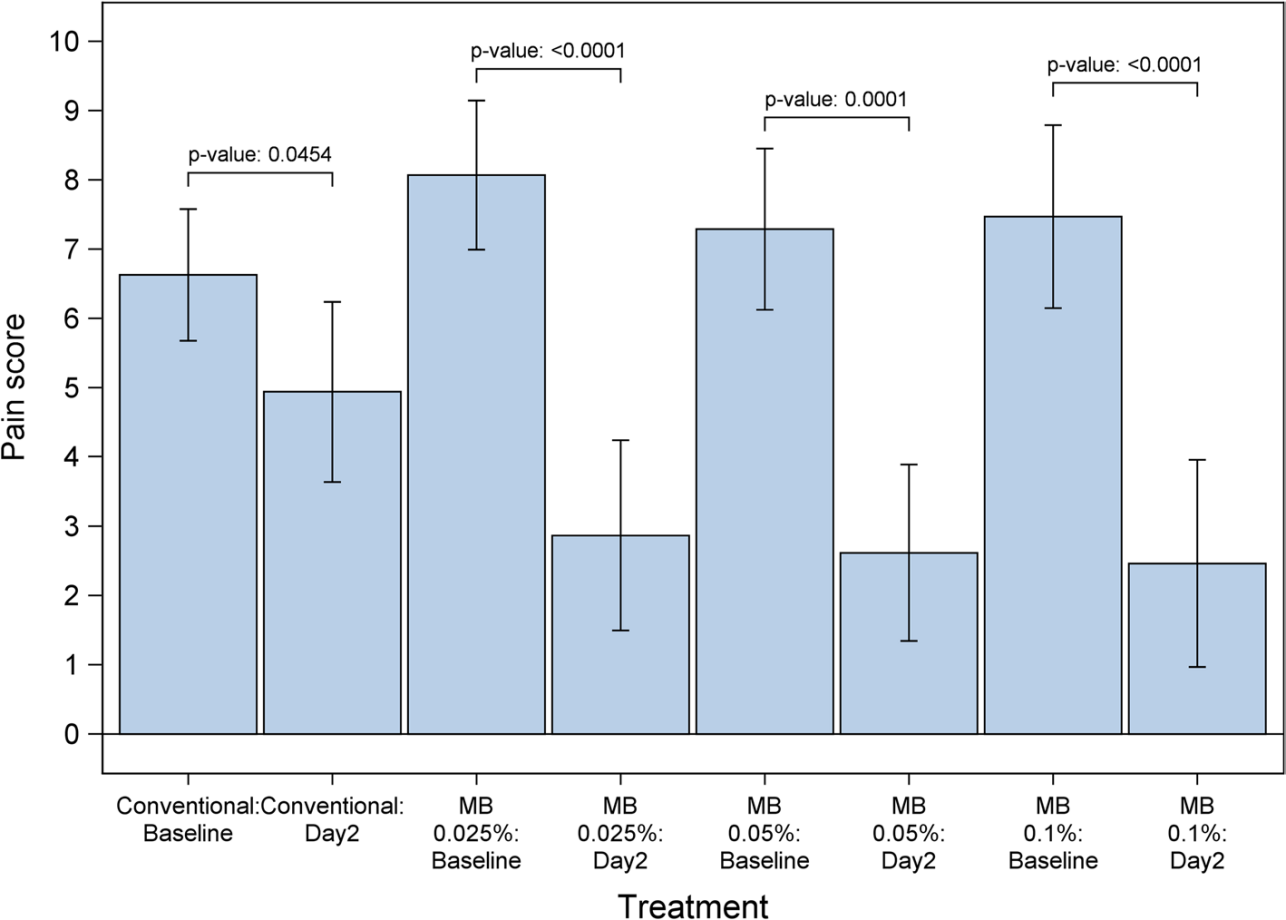
Mean pain numeric rating scale score reductions from baseline to day 2 by treatment arm. Mean pain numeric rating scale scores before and after treatment are shown for patients who received conventional therapy alone and those who received conventional therapy plus methylene blue (MB) oral rinse at three different concentrations. Lines indicate 95% confidence intervals of the mean

Among the 60 patients who completed the trial, 16 received CTx alone, 15 received CTx+MB 0.025%, 14 received CTx+MB 0.05%, and 15 received CTx+MB 0.1%. After patients began receiving the MBOR, a rapid reduction in pain scores was observed within the first 24 h, with additional improvement at 48 h (i.e., baseline NRS score minus NRS score on day 2): mean ± SD changes in pain NRS scores at day 2 were 5.2 ± 2.81 for CTx+MB 0.025%, 4.54 ± 2.93 for CTx+MB 0.05%, and 5.15 ± 2.64 for CTx+MB 0.1%, compared with 1.69 ± 3.09 for CTx alone (Table 1). Tukey pairwise comparisons indicated that the differences were significant for the three arms of CTx+MB compared with the CTx alone arm (*P*=0.0071, *P*=0.0506, *P*=0.0114 Figs. 2 and 3).

**Fig. 4:**
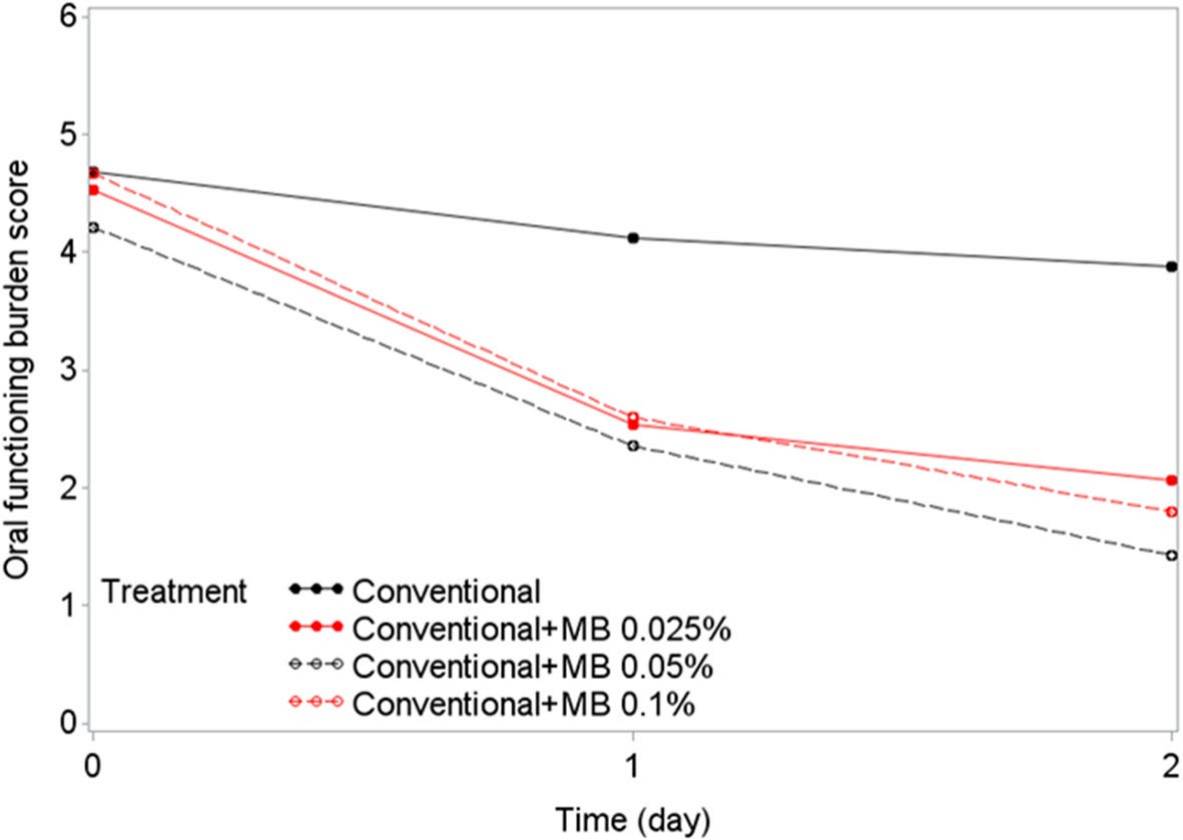
Changes in oral functioning burden scores over the first 2 days of treatment. Mean oral functioning burden scores at day 2 after the start of treatment were significantly better in patients who received conventional therapy plus methylene blue (MB) oral rinse at three different concentrations than in patients who received conventional therapy alone (Tukey pairwise comparison; (*P* = 0.0171, *P* = 0.0038, *P* = 0.0019)

**Fig. 5:**
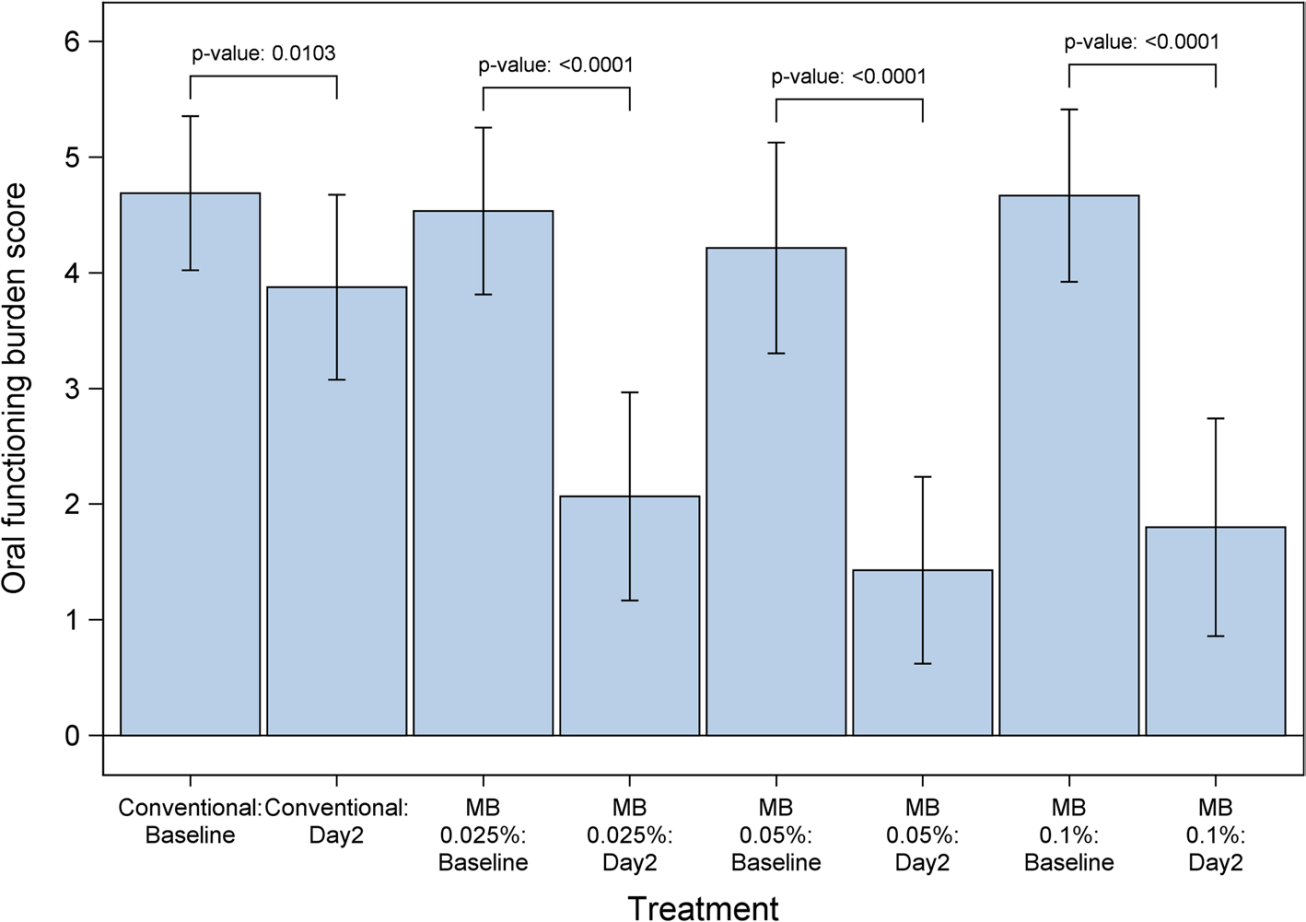
Mean oral functioning burden score reductions from baseline to day 2 by treatment arm. Mean oral functioning burden scores before and after treatment are shown for patients who received conventional therapy alone and those who received conventional therapy plus methylene blue (MB) oral rinse at three different concentrations. Lines indicate 95% confidence intervals of the mean

Similarly, a rapid reduction in OFB scores was observed within the first 24 h, with additional improvement at 48 h (i.e., baseline OFB score minus OFB score on day 2): mean ± SD changes in OFB scores on day 2 were 2.47 ± 1.55 for CTx+MB 0.025%, 2.79 ± 1.72 for CTx+MB 0.05%, and 2.87 ± 1.60 for CTx+MB 0.1%, compared with 0.81 ± 1.11 for CTx alone (Table 1). Tukey pairwise comparisons indicated that the differences were significant for all three MB arms compared with the CT alone arm (*P*=0.0171, *P*=0.0038, *P*=0.0019 Figs. 4 and 5).

Although the maximum pain relief was reported within minutes of the first dose in most patients who received MB (*n* = 34/44, 77%), almost all patients required several doses—up to 6 (48 h)—to achieve sustained pain relief. Similarly, in more than half of the patients who received MB (*n* = 25/44, 57%), pain recurred between 4 and 8 h (*n* = 21/44, 48%). However, the intensity of recurring pain was reported at lower scores than at the baseline. The trial had sponsored only 100 mL of the MB solution, but we found that more than half of the patients who received it (*n* = 26/44, 59%) requested to continue the MB oral solution after day 2. Two patients required only one dose of MB for complete pain relief.

Although we aimed to determine the MEDD variations for each patient, it was challenging to differentiate the doses of opiate analgesics before and after treatment with MBOR. Most patients had additional indications for the use of systemic analgesics; therefore, we could not draw the impact of MBOR on opiate use (Table 1).

### Association between MB dilutions and efficacy

The main reduction in pain NRS and OFB scores was evident within the first 2 days, with the most substantial effect observed on the first day of use of MB. Although the MB concentration of 0.1% appeared to improve OFB more effectively than the 0.05% and 0.025% concentrations, there was not a statistically significant difference between each pair of these groups (*P* = 0.9401, *P* = 0.8848, *P* = 0.9989, Tukey pairwise comparison). Similarly, the reduction in pain NRS scores on day 2 was not statistically different between three concentrations of MB (*P* = 0.9299, *P* = 1.0000, *P* = 0.9477, Tukey pairwise comparison).

### Adverse events

A few mild, transient, and self-resolved events were reported. Five patients experienced oral burning sensation during the first use, including one patient who subsequently discontinued the therapy. In addition, three other patients discontinued using MBOR (two claimed no pain relief, and one did not provide a justification). Transient mouth and teeth discoloration were reported, but this resolved with oral hygiene or after meals. No permanent stain or other side effects were reported at the 30-day follow-up.

## Discussion

Our phase 2 randomized clinical trial showed that MBOR led to significantly better pain reduction and improved OFB than CTx alone, with minimal, transient, and self-resolved adverse events. No events were reported at 30-day follow-up.

MB has been used to treat various painful syndromes, including postoperative pain, discogenic pain, and neuropathic pain [20–22].

More than 75% of patients reported analgesia within minutes of the first dose in our study. Although most patients required several doses, a significant reduction in pain NRS scores and improvement in OFB was observed in the first 24 h, with additional improvement at 48 h. After 2 days, 59% of patients still required treatment. But no patients required treatment after 7 days. The time course of MB analgesia seems to support a neurolysis mechanism. MB seems to be a long-term inhibitor of peripheral axons by denaturation of free nociceptive nerve endings. A typical peripheral nerve injury initially leads to acute axonal degeneration within 30 min [23]. The degeneration process continues with swelling of the cell membrane and eventually disrupts myelin sheaths in 24 h in the peripheral nerve system [24]. Upon observing insensitive skin for up to 1 month following intracutaneous injection of MB, Rygick proposed that MB had a neurolytic effect [25]. Eusebio et al. also supported the neurolysis theory, reporting absent distinct nerve endings on perianal skin from patients treated with intracutaneous MB [26]. The onset of analgesia may also be correlated with the severity of mucositis or nerve exposure, allowing more direct contact with MB.

Other proposed mechanisms of MB analgesia are anti-inflammatory, by inhibition of nitric oxide inflammatory pathway–via induced guanylate cyclase [27–29]. Miclescu et al. observed a decrease in tactile allodynia with systemic MB in patients with refractory neuropathic pain [30]. Phosphorylation or blockade of the N-methyl-D-aspartate (NMDA) receptors in the dorsal horn leads to postsynaptic changes in second-order neurons, which are commonly manifested as allodynia [31]. MB decreases guanylate cyclase and NO synthesis, which in turn inhibits NMDA receptor activation [32–34]. However, NMDA antagonism requires systemic MB and occurs at the spinal cord level. Hence, the NO synthesis route does not support analgesia via the direct contact route.

Whereas several therapies for the treatment of oral pain associated to mucositis have their shortcomings, even a diluted topical MB solution (0.025%) can be highly effective as it can readily reach the target nerves with a minimum barrier. We observed no statistical difference in analgesia between the three MB concentrations, our results support using the most diluted solution initially to further reduce the transient discoloration and perhaps the burning sensation.

Unlike other oral rinses currently used in clinical practice, MBOR does not result in local anesthesia of the oral cavity; it therefore does not change the perception of the taste or inhibit the gag reflex. In addition, MB demonstrated an accumulative analgesic effect after multiple doses. Although the present showed good results, the trial was limited in that it was not a placebo-controlled study. The study recruited a relatively small number of patients. We acknowledge that not discontinuing CTx in patients assigned to receive MB could result in a co-intervention bias.

## Conclusions

This clinical trial demonstrated effectiveness in pain relief and improving OFB using MB + CTx compared with CTx alone. This trial of MBOR supports its use as a low-risk, efficient, and easy-to-use treatment for refractory oral pain due to OM from cancer therapy. Whereas MBOR is not commercially available, it must be compounded individually, that in some cases led to costs too great for some individuals that remains a challenge to overcome. However, the low cost and wide availability of MB makes it potentially accessible to patients of all socioeconomic backgrounds widely worldwide. Further studies are needed to evaluate the possible effect of MBOR in OM severity and healing time.

## Supplementary Information

**Additional file 1.**

## Data Availability

For original data repositories, please contact croldan@mdanderspon.org. Raw data was submitted with the manuscript.

## Abbreviations

OM: Oral mucositis
MB: Methylene blue
MBOR: Methylene blue oral rinse
CTx: Conventional therapy
NRS: Numeric rating scale
OFB: Oral functioning burden
MEDD: Morphine equivalent daily dose
NMDA: N-methyl-d-aspartate
